# Is there a link between Spigelian and inguinal hernias? A case series

**DOI:** 10.1007/s10029-024-03061-5

**Published:** 2024-06-27

**Authors:** R. Lorenz, U. Vollmer, J. Conze, F. Loch, K. Paul-Promchan, R. Mantke, C. Paasch, R. Wiessner

**Affiliations:** 1 Hernia Center, 3+CHIRURGEN, Klosterstrasse 34/35, 13581 Berlin, Germany; 2 Department of General and Abdominal Surgery, Clinic for General and Abdominal Surgery, Medical University Brandenburg an Der Havel, Fehrbelliner Strasse 38, 3+CHIRURGEN, Klosterstrasse 34/35, 13581 Berlin, Germany; 3 Havelklinik Berlin, 3+CHIRURGEN, Klosterstrasse 34/35, 13581 Berlin, Germany; 4 Park-Klinik Weißensee, Department of General Surgery, Schönstrasse 80, 13086 Berlin, Germany; 5 UM Hernienzentrum Dr. Conze, Arabellastraße 17, 81925 Munich, Germany; 6 Department of Surgery, Charité—Universitätsmedizin Corporate Member of Freie Universität Berlin and Humboldt-Universität Zu Berlin, Hindenburgdamm 30, 12203 Berlin, Germany; 7 Department of General Surgery, Hospital Bethel Berlin, Promenadenstrasse 3-5, 12207 Berlin, Germany; 8 Faculty of Medicine, Brandenburg Medical School Thedor Fontane, Brandenburg, Germany; 9 Faculty of Health Sciences Brandenburg, Brandenburg Medical School Theodor Fontane, Brandenburg an Der Havel, Germany; 10 Bodden-Klinik Ribnitz-Damgarten, Sandhufe 2, 18311 Ribnitz-Damgarten, Germany

**Keywords:** Spigelian hernia, Ventral hernia, Inguinal hernia, Case series

## Abstract

**Introduction:**

Spigelian hernias are among the rare primary ventral hernias. Diagnosis is often difficult, as many cases are asymptomatic. Spigelian and inguinal hernias are usually considered separately in current scientific literature. With this case series, we want to illustrate a possible relationship between the neighboring hernia types.

**Methods:**

In this article, we report on a case series of Spigelian hernias that were operated on in five hernia centers in the period from January 1st, 2021 to October 31st, 2023. We have summarized all patient characteristics with previous operations and the result of the secondary operation.

**Results:**

We report a case series with 24 Spigelian hernias, 15 of which have a connection to previous inguinal hernias. In these cases, however, it is not certain whether these are primarily overlooked or occult hernias or whether these Spigelian hernias have arisen secondarily, as a result of previous hernia surgery.

**Summary:**

With this case series, we would like to point out a possible connection between Spigelian hernia and inguinal hernia. Further studies are needed to shed more light on this entity and explain its genesis.

## Introduction

Spigelian hernias are among the rare abdominal wall hernias. They are named after the Flemish anatomist, surgeon and botanist Adrian van den Spieghel (1578–1625). The first description of a Spigelian hernia in 1764 goes back to Thaddeus Klinkosch, a Czech anatomist [1–3].

The term “Spigelian hernia” is used for congenital or acquired defects in the intersection of linea semilunaris and linea arcuata where the fascia of the internal oblique and the transverse abdominal muscles form the spigelian aponeurosis. Through these defects preperitoneal fatty tissue or a peritoneal sac containing parts of omentum majus or intestine can protrude. Overall, the incidence must be considered low, but exact figures are lacking. Women, mainly in the 4th to 7th decade of life, are probably affected more often than men [3].

The genesis of these Spigelian hernias is still not fully understood. They are always located in the linea semilunaris, which must be described as "locus minoris resistentia" [4]. Predisposing factors are patients over 50 years of age, obesity, chronic obstructive pulmonary disease (COPD) and the existence of other abdominal wall hernias [5].

In literature, case reports and case series predominate. Only five reviews with small numbers of patients (10 to 107) are available [6–10]. A recent database study from Denmark summarized 365 cases, with 16.4% being emergency interventions [11]. A meta-analysis does not exist, yet. Since 2009 there has been a classification of primary ventral hernias, including Spigelian hernias, by the European Hernia Society. They are divided into small (< 2 cm), medium (≥ 2–4 cm) and large (≥ 4 cm) [12]. In 2020 an expert group of the European and Americas Hernia Society published a review of the existing literature and a guideline for ventral hernias in rare circumstances. [3].

Diagnosis is sometimes difficult, as a large proportion of cases remain asymptomatic for a long time. 25% of cases are not diagnosed until an emergency procedure [3].

To date, Spigelian and inguinal hernias are considered separately and a connection between these two types of hernias has not yet been demonstrated. In a personal series, we have recently been able to observe that some Spigelian hernias have had a previous inguinal hernia operation.

The aim of this case series is to visualize a possible connection between Spigelian and inguinal hernias, to point out a possible coincidence and to develop awareness for this combination in some cases with unclear symptoms.

## Methods

We conducted a retrospective case series of patient data from five specialized hernia centers in Germany (3 + CHIRURGEN Hernia center Berlin, Hospital Bethel Berlin, Park-Klinik Weißensee Berlin, UM Hernia centre Dr. Conze in Munich and Bodden-Klinik Ribnitz-Damgarten). In the period from January 1st, 2020 to October 31st, 2023, 24 cases of Spigelian hernias were operated. All patients had a symptomatic Spigelian hernia with exercise-dependent swelling and discomfort and/ or pain that led to surgery. All of them have consented to the publication as a case series.

Preoperative diagnostics included a detailed medical history including all previous hernia operations, clinical examination, dynamic ultrasound and, in selected cases, an additional computed tomography or magnetic resonance imaging (MRI). All cases were examined clinically by the surgeons four weeks postoperatively to record the early postoperative outcome. In the analysis we recorded the master data of the patients and the existing data of the previous inguinal or ventral hernia operation including date, hernia classification and operation technique. Regarding the surgical treatment of the Spigelian hernia, the size of the hernia, the operation technique, the size of mesh, the duration of the operation, the intraoperative and postoperative complications up to four weeks were recorded.

### Statistics

For the descriptive analysis of the case series we have used Microsoft Excel. No multivariate or univariate analysis was conducted. In addition, we carried out a photo documentation of selected cases after agreement of patients to visualize representative examples.

## Results

**Fig. 1:**
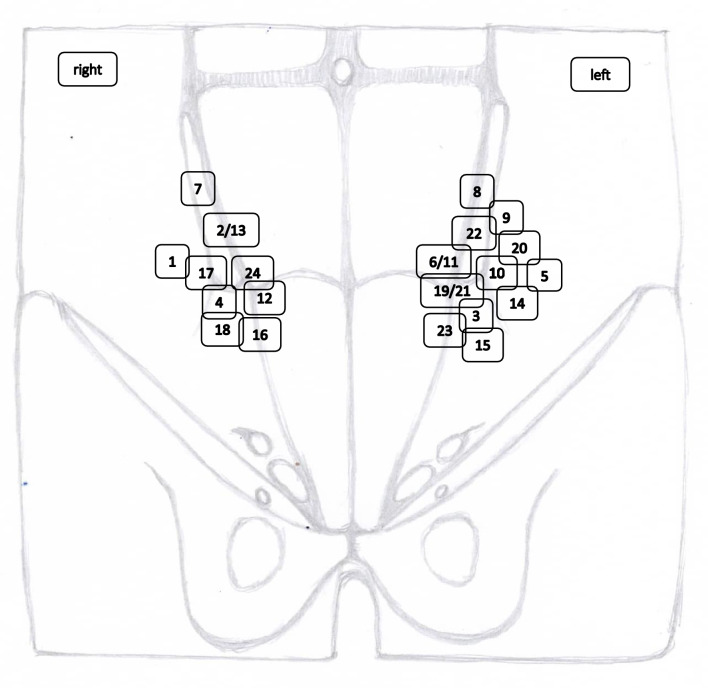
Spigelian Hernia localisations on the abdominal wall with case numbers

All together 24 Spigelian hernias were operated on between January 1st, 2020 to October 31st, 2023 (12 female and 12 male). The exact location of all Spigelian hernias was marked in a schematic drawing (Fig. 1). The age was on average 68.7 years (range 46–93 years, *n* = 24). Patient characteristics and medical history are summarized in Table 1. A total of 58% (*n* = 14) of the Spigelian hernias are medium-sized hernias according to EHS Classification, 17% are classified as large, and 25% as small Spigelian hernias [12].

**Table 1:** Cases with patient characteristics and medical history

		Age	BMI (kg/m^2^)	Smoking > 10 cigarettes/d	Previous inguinal/ventral hernia or abdominal operation-date	Time interval inguinal—spigelian hernia operation (months)	Previous inguinal/ventral hernia operation-technique	Previous inguinal/ ventral hernia operation-classification (EHS-Classification)	Previous inguinal hernia operation-side	Number of previous Hernias
1	F	56	27.9	+	05/2020	19	Inguinal—Gilbert	L III	Right	2
2	M	80	25.6	+	08/2020	15	Inguinal—TEP	L II	Right	1
3	F	49	34.8	+	07/2021	2	Inguinal—TEP	L II	Left	2
4	M	93	24.7	−	06/2022	12	Inguinal—Gilbert	C III L III M II	Right	3
5	M	57	29.6	+	09/2009	160	Inguinal—Gilbert	M III	Left	1
6	M	60	24.0	+	05/2022	13	Inguinal—TIPP	M III	Left	3
7	M	56	22.2	−	09/2022 Abdominal Trauma	−	No	−	−	0
8	F	88	21.9	−	2007, 07/2013	123	Inguinal—Lichtenstein	L III	Bilateral	4
9	F	73	25.0	−	11/2020 Lap. Hysterectomy	−	No	−	−	0
10	M	73	24.3	−	02/2012	141	Inguinal—Gilbert	C III L II M II	Bilateral	5
11	M	77	25.4	−	1952, 2013, 05/2023	1	Inguinal TAPP, Ventral IPOM	MIII, R1	Left	3
12	F	46	27.3	−	05/2020 lap. Hysterectomy	−	No	−	−	0
13	F	88	25.7	−	1993, 2015, 05/2023 (+ CCE)	−	Ventral—IPOMplus	M2–M4, W1	−	2
14	F	72	41	+	03/2017	48	Inguinal—TAPP	M II	Left	2
15	M	64	28	−	No	−	No	−	−	0
16	M	48	36	−	05/2022	18	Inguinal—eTEP	M III	Bilateral	1
17	M	50	29.3	+	2017	−	Umbilical—PUMP	−	−	1
18	M	81	26.5	+	12/1970	625	Inguinal—Pure Tissue	unknown	Left	1
19	F	58	24.2	−	03/2019	44	Inguinal—TAPP	L II	Right	1
20	F	73	34.3	−	2018	−	Hiatal hernia	−	−	1
21	F	80	25.4	−	No	−	No	−	−	0
22	F	54	30.1	+	09/2021	2	Inguinal—TEP	L II	Bilateral	2
23	F	83	27.2	−	1990, 10/2008	−	Ventral—Suture, Ventral—Sublay	M4, W2	−	3
24	M	89	24.2	−	10/2005	193	Inguinal-Pure Tissue	unknown	Bilateral	2

European Hernia Classification For Inguinal Hernias: *C* combined, *F* femoral, *L* lateral = indirect, *M* medial = direct, Size I < 1,5 cm, II 1,5-3cm, III > 3cm European Hernia Classification For Ventral Hernias: M2 = Midline epigastric, M3 = Midline umbilical, M4 = Midline hypogastric, W = Width W1 =  < 4cm, W2 4- < 10cm

*BMI* Body mass index

Operation techniques: *PUMP* Preperitoneal umbilical mesh plasty, *TEP* Total extraperitoneal plasty, *eTEP* Extended totally extraperitoneal repair, *TAPP* transabdominal preperitoneal plasty, *IPOM* intraperitoneal onlay mesh plasty, *CCE* cholecystectomy, *Gilbert* Bi-Layer Mesh (anterior and posterior mesh placement with a specific device), *TIPP* transinguinal preperitoneal plasty

In 15 of the 24 documented patients with a Spigelian hernia, we found a history of a previous inguinal hernia repair on the same side. The average interval between the inguinal and Spigelian surgery was 94.4 months (range 1 to 625 months, *n* = 15). In 7 of these 15 cases, a Spigelian hernia developed within 24 months of inguinal hernia surgery (47%, *n* = 15). One case of Spigelian hernia was obviously already present during the procedure for the inguinal hernia.

In the majority of cases, the previous inguinal hernia was surgically treated openly or endoscopically with a posterior mesh position.

In the Spigelian hernia repair, an open preperitoneal mesh procedure dominated. The average operating time was 68 min (range 25–105 min, *n* = 24) The pre-existing inguinal mesh was not removed in any case (Table 2). The body mass index of the patients was 27.7 kg/m^2^ (range 21.9—41.0 kg/m^2^, *n* = 24). All patients had a median of 1.5 hernia operations in their history (range 0–5, *n* = 24). A total of 37.5% of the patients smoked more than 10 cigarettes per day.

**Table 2:** Cases with Operation results of the Spigelian Hernia operation

	Spigelian hernia site	Incarcer	Spigelian hernia, OP-date	Spigelian OP-technique	Spigelian hernia-size (cm)	Spiegelian EHS-classification	Spigelian mesh-size (cm)	OP min	Hosp. stay	Mesh removal	IOP Complic	POP Complic. (30d)
1	R	−	12/2021	Open PMP	2 × 3	L M	9 × 9	69	2	No	No	No
2	R	−	11/2021	Open PMP	3 × 3	L M	10 × 10	73	1	No	No	Seroma
3	L	−	09/2021	Open PMP	3 × 4	L L	10 × 10	84	2	No	No	No
4	R	−	06/2023	Open PMP	2 × 2	L M	9 × 9	52	2	No	No	No
5	L	−	06/2023	Open PMP	1,5 × 1,5	L S	8 × 8	34	2	No	No	No
6	L	−	06/2023	Open PMP	2 × 2	L M	8 × 8	75	2	No	No	No
7	R	−	06/2023	Open PMP	2 × 3	L M	9 × 9	76	1	−	No	No
8	L	−	10/2023	Open PMP	2 × 2,5	L M	9 × 9	40	2	No	No	No
9	L	−	11/2023	Open PMP	4 × 5	L L	15 × 15	85	2	No	No	No
10	L	−	11/2023	Open PMP	1 × 1,5	L S	5 × 6	48	1	No	No	No
11	L (and incisional hernia	−	05/2023	Lap. IPOM +	2 × 2 (2 × 3 incisional Hernia)	L M	20 × 15	60	4	No	No	No
12	R	−	05/2020	Lap. IPOM +	2 × 2	L M	15 × 15 round	45	3	No	No	No
13	R	−	05/2023	Lap. IPOM +	3 × 5	L L	12 × 12 round	55	4	No	No	No
14	L	−	03/2021	Lap.IPOM	2 × 3	L M	15 × 15 round	50	6	No	No	No
15	L	−	11/2023	Open PMP	1,5 × 2	L M	8 × 8 round	26	2	No	No	No
16	R	−	11/2023	Open PMP	2 × 3	L M	8 × 8 round	42	2	No	No	No
17	R	−	03/2023	TEP + Inguin. hernia	1 × 1	L S	10 × 15	66	2	No	No	No
18	R	+	01/2023	Tissue repair	2 × 2	L M	No	42	3	No	No	No
19	L	−	11/2022	TEP + Inguin. hernia	1 × 1	L S	10 × 15	45	1	No	No	No
20	L	−	06/2022	Open PMP	4 × 5	L L	10 × 15	105	6	No	No	No
21	L	−	09/2021	TEP + Inguin. hernia	1 × 1	L S	13 × 17	40	1	No	No	No
22	L	−	11/2021	Tissue repair	2 × 2	L M	No	25	2	No	No	No
23	L	−	04/2021	Open PMP	1 × 1,5	L S	10 × 15	60	6	No	No	No
24	R	+	11/2021	Tissue Repair	2 × 2	L M	No	40	10	No	No	No

*Open PMP* Open preperitoneal mesh plasty, *Lap. IPOM +* Laparoscopic IntraPeritoneal Onlay Mesh plasty plus Augmentation of the defect, *TEP* Total Extraperitoneal mesh Plasty

The size of the hernia gap was 5.97 cm^2^ (range 1–20 cm^2^). The repair of the hernia was sutured in three cases; otherwise meshes ranging in size from 5 × 6 cm to 15 × 20 cm were used. The hospital stay of the patients was 2 days (range 1—10 days, *n* = 24). Only one patient developed a seroma postoperatively, which was treated conservatively (Table 2).

In this article we collected and summarized selected cases:

- Fig. 1: exact location of 24 spigelian hernias
- Figs. 2,3: preoperative view on the Abdominal wall in selected cases
- Figs. 4,5: CT—scan of selected spigelian hernias
- Figs. 6, 7, 8, 9, 10: intraoperative findings and repair steps with mesh insertion in selected cases

**Fig. 2:**
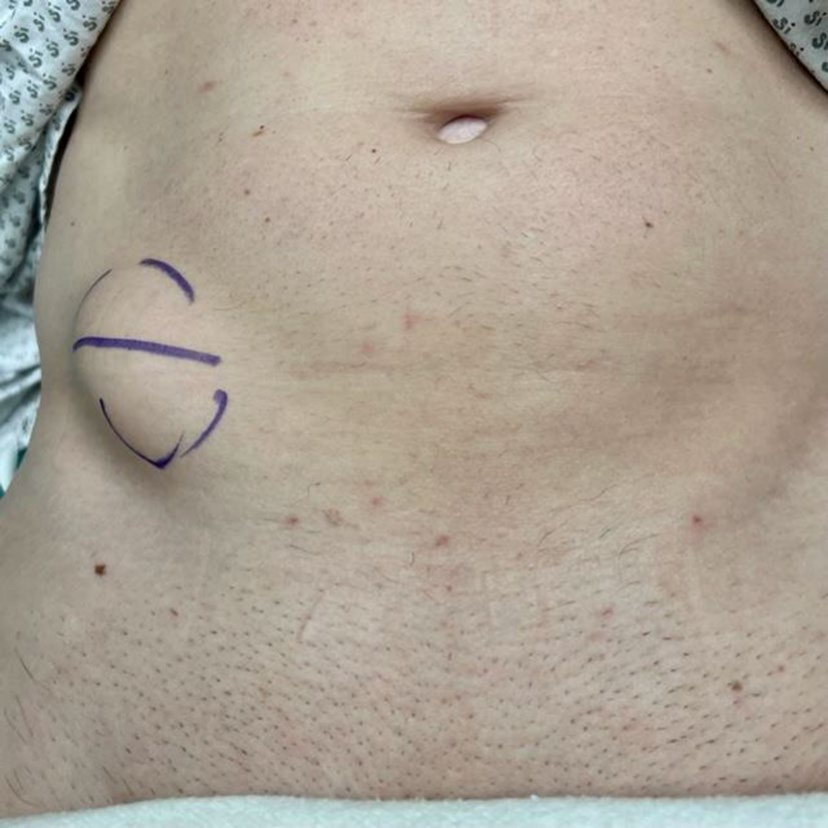
Selected preoperative picture of right spigelian Hernia (Foto © R. Lorenz)

**Fig. 3:**
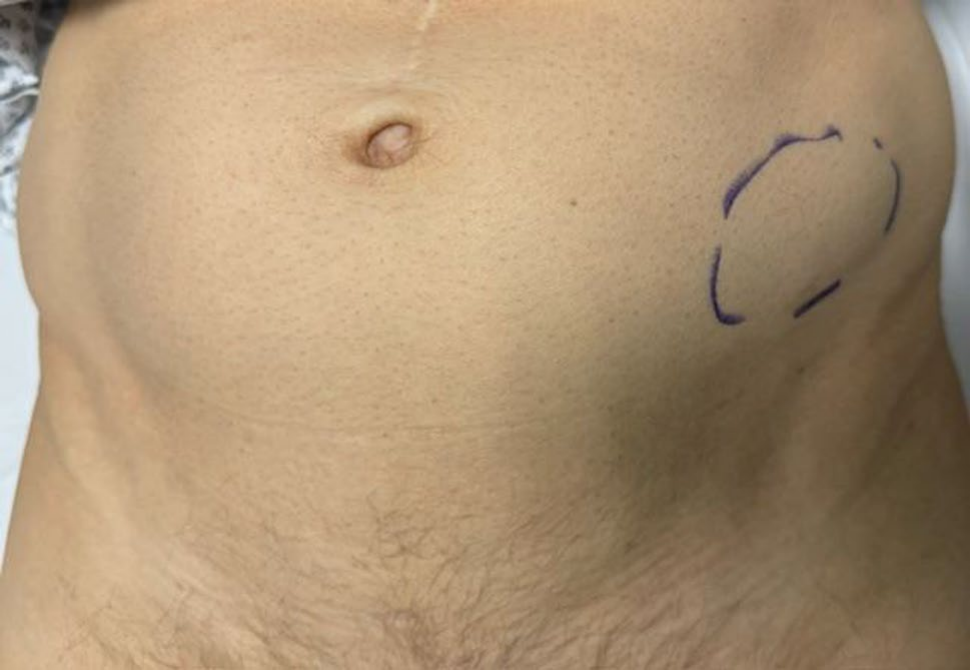
Selected preoperative picture of left spigelian Hernia (Foto © R. Lorenz)

**Fig. 4:**
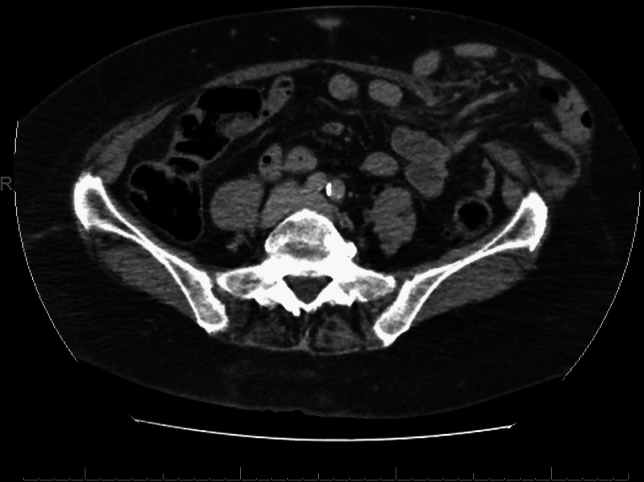
CT-Scan of left spigelian Hernia (Foto © U. Volmer)

**Fig. 5:**
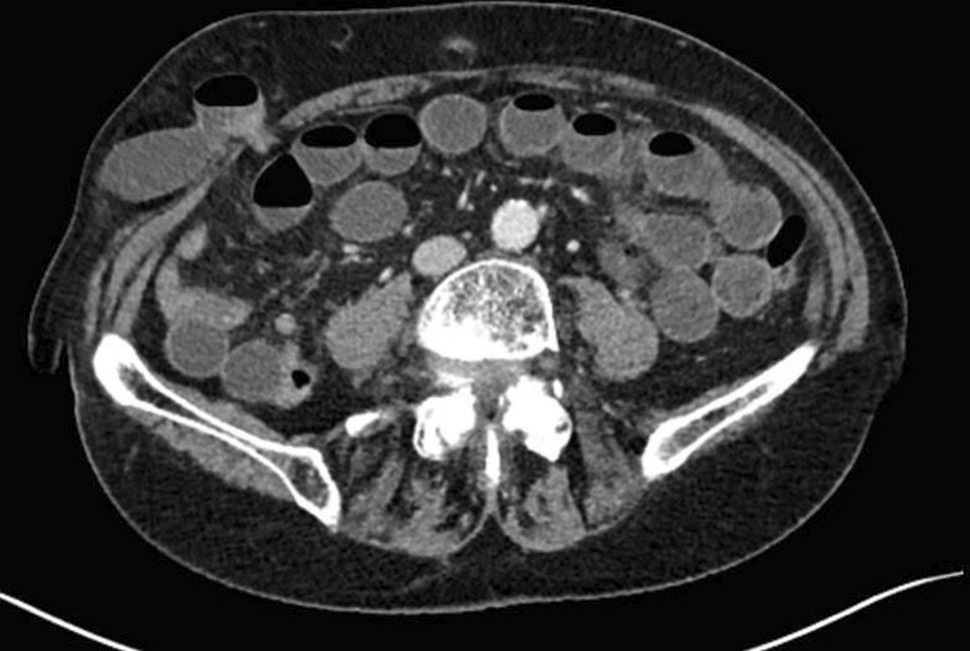
CT-Scan of right spigelian Hernia (Foto © U. Volmer)

**Fig. 6:**
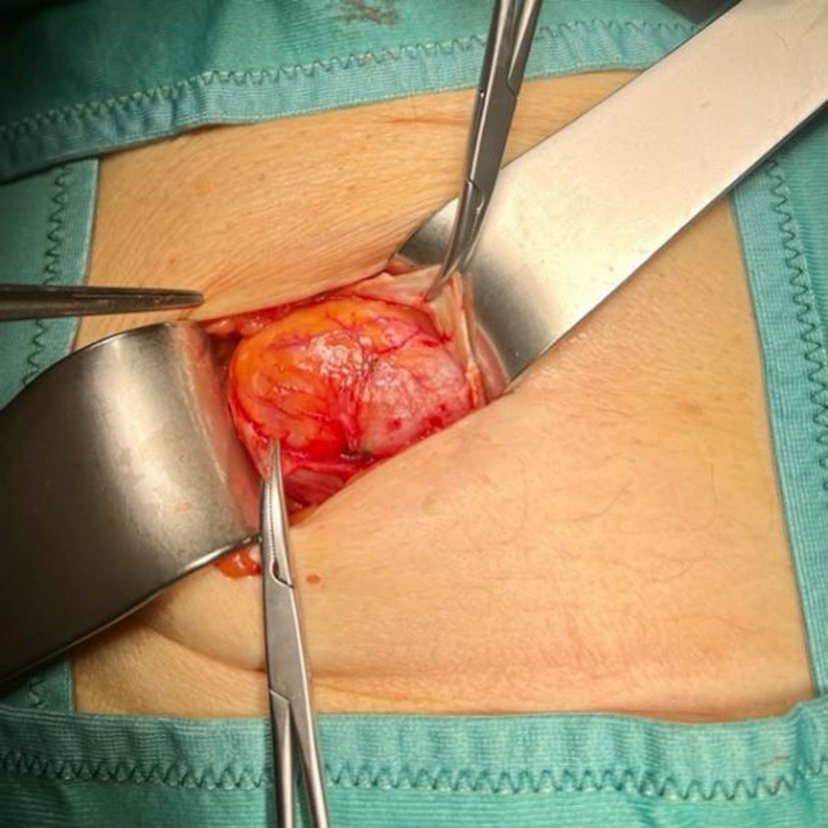
Selected intraoperative picture with spigelian hernia sac (Foto © R. Lorenz)

**Fig. 7:**
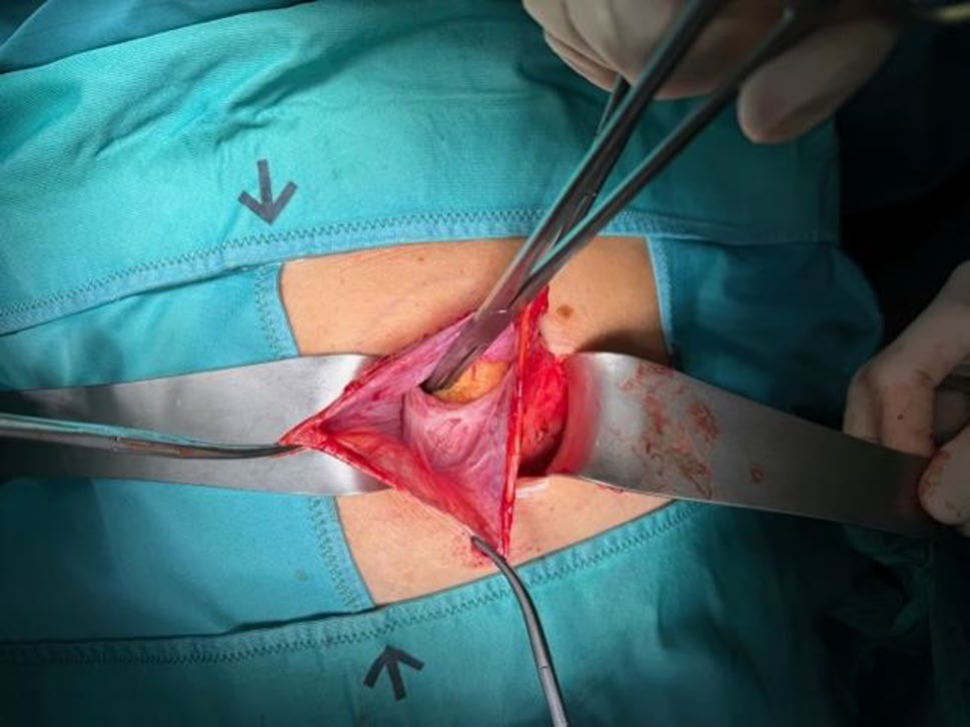
Selected intraoperative picture with spigelian hernia sac (Foto © R. Lorenz)

**Fig. 8:**
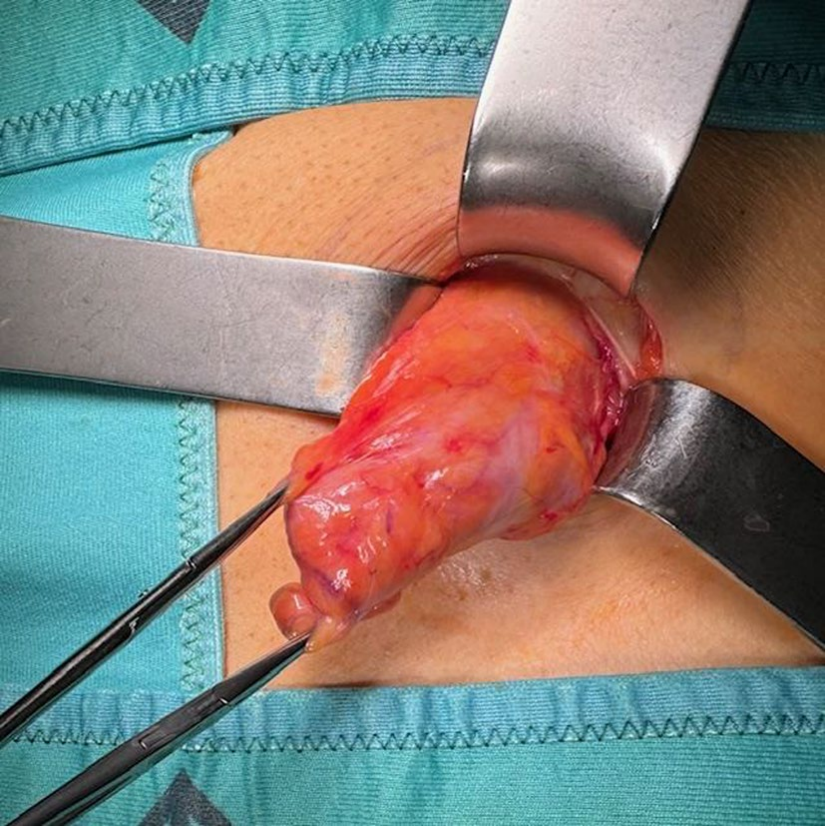
Selected intraoperative picture with spigelian hernia sac (Foto © R. Lorenz)

**Fig. 9:**
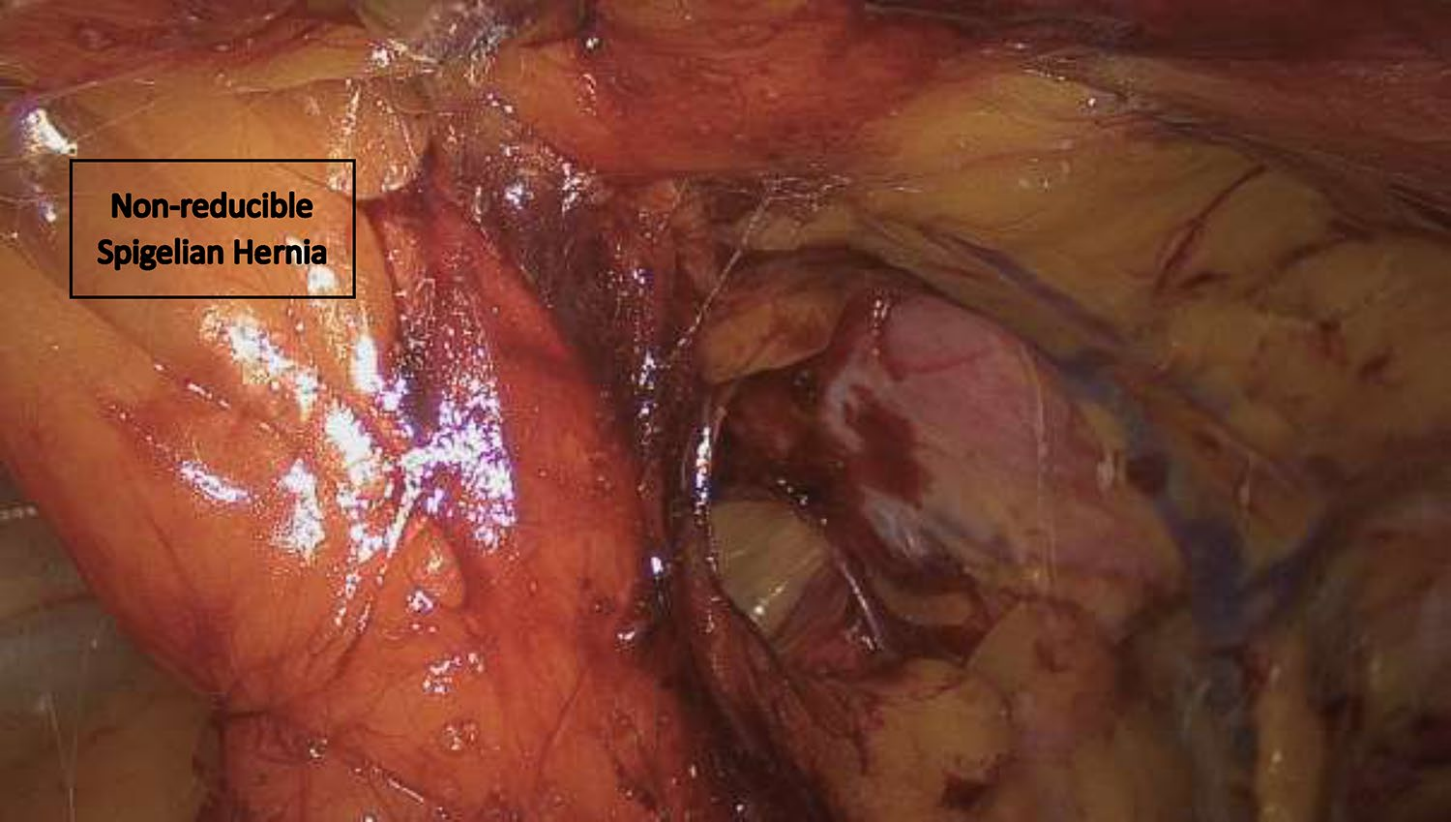
Endoscopic view on non-reducible spigelian hernia (Foto © U. Volmer)

**Fig. 10:**
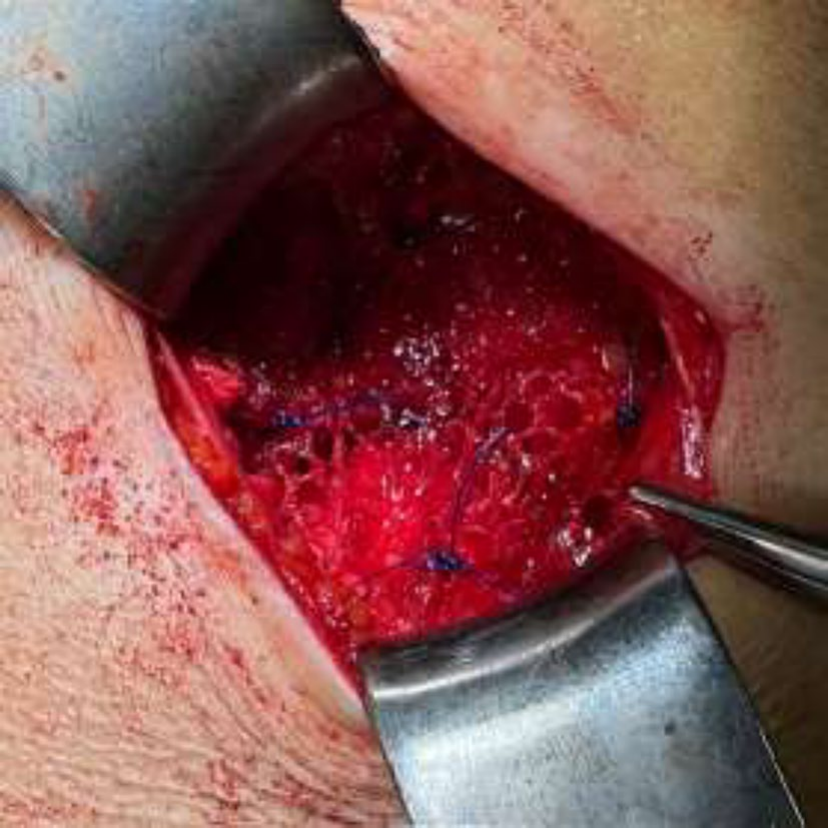
Selected intraoperative picture with mesh insertion in spigelian hernias (Foto © R. Lorenz)

## Discussion

In our case series, 15 of the 24 patients with Spigelian hernia had undergone a previous inguinal hernia operation. In almost half of these 15 cases, a Spigelian hernia developed within 24 months of previous inguinal hernia operation. In one case it was apparently overlooked during the primary inguinal hernia operation as the patient continued to be clinically symptomatic. This direct relationship has not yet been discussed in the scientific literature. Recently, Weijie et al. described the possible correlation of Spigelian hernias combined with inguinal hernias, as “Spigelian inguinal complex” [13]. He postulated that such a combination is not uncommon.

In one of the 24 cases, a post-traumatic Spigelian hernia developed (or became symptomatic) after blunt abdominal trauma.

Not all of the Spigelian hernias summarized in our case series were localized in the typical Spigelian belt (Fig. 1). Some publications support a wider anatomical definition [14, 15]. Some of these cases could also be referred to as interstitial hernias or muscular groin hernias, although this type of hernia has not yet been scientifically confirmed [16–19].

Against this background, diagnostics are given a particularly high priority. The sensitivity of the dynamic ultrasound examination is estimated to be high [20]. In selected cases, an MRI or dynamic MRI can help to differentiate. In cases without a peritoneal bulge, detection of the defect can be difficult in laparoscopic surgery; likewise in open surgery in cases of interstitial hernias with an intact external aponeurosis.

The association between Spigelian hernias and inguinal hernias is striking, especially since the regions are adjacent. Various possible scenarios have been described in the literature:

- Are they overlooked occult hernias [21]?
- Is there a confusion of inguinal and ventral hernias and Spigelian hernias?
- Are there any pathophysiological changes in the abdominal wall after an inguinal or ventral mesh repair that may lead to instability in the immediate surrounding of the mesh area as a result of shear forces [22]?
- Is there a congenital “Spigelian-inguinal complex“ [13, 23]?

Addendum 1: Occult Spigelian hernias associated with laparoscopies were first reported by Pajaanen in 2006. The overall frequency of unexpected Spigelian hernias was 5 of 201 (2%). [21].

Addendum 2: The literature also reports on hernias that appeared as Spigelian hernias, which were then ultimately identified intraoperatively as lateral hernias [24–26].

Addendum 3: The third thesis would also be supported by the case report on the development of a Spigelian hernia after laparoscopic incisional hernia surgery [22]. We also had a case in the immediate neighborhood of an IPOM mesh inserted 8 years earlier (Table 1, Case 13). This could be caused by scar shrinkage of the mesh area. In the initial phase mesh area shrinkage was observed in up to 33% of cases over time after surgery with synthetic meshes [27, 28]. However, these shrinkages have not been confirmed by other authors [29, 30]. Traumatization of the arcuate line through the lateral approach during endoscopic or open preperitoneal operations could play a role here.

Addendum 4: In some case studies there seems to be a congenital relationship between Spigelian hernias and cryptochism in newborns and young children, [25, 31–33]. In addition, the occurrence of multiple hernias could generally be attributed to a systematic metabolic disorder. Specifically, it is assumed that an abnormal expression of metalloproteinases can lead to a degradation of the extracellular matrix and thus to an altered ratio of collagen 1 and 3 [34]. Especially the substantial rate of smokers in our study might contribute to a metabolic disorder with subsequently wound healing disorder [34]. More research on that topic is needed.

One case in this case series describes the development of a traumatic Spigelian hernia as a result of blunt abdominal trauma. This case may be of interest for understanding the genesis of Spigelian hernias. Individual case reports of traumatic Spigelian hernias already exist, but they are considered very rare overall [35]. Though it remains uncertain whether the defect developed due to the trauma or became symptomatic in a pre-existing defect. Further case reports and scientific research on the anatomy and physiology of the abdominal wall are needed to confirm this correlation [36]. Maybe register data could also be accessed in the future to be able to prove a correlation between Spigelian and inguinal hernias.

## Limitations

These observations come from the working group with five participating hernia centers. Of course, these can be random observations and are not representative and statistically relevant due to the small number of cases. Not all Spigelian hernias summarized in this case series are to be understood as such according to the current definition. However, there is no clear way to assign these cases correctly.

## Summary

The knowledge on Spigelian hernias is still quite limited. In this observational case series, a coincidence or even a possible correlation between the appearance of a Spigelian hernia after inguinal mesh repair is demonstrated and discussed. A careful and precise diagnostic with more awareness for spigelian hernias is warranted, especially after previous inguinal mesh repair. Further research on that topic is necessary?

## Data Availability

Data are available on request.
